# CRISPR-Cas9-Based Mutagenesis of the Mucormycosis-Causing Fungus *Lichtheimia corymbifera*

**DOI:** 10.3390/ijms21103727

**Published:** 2020-05-25

**Authors:** Sandugash Ibragimova, Csilla Szebenyi, Rita Sinka, Elham I. Alzyoud, Mónika Homa, Csaba Vágvölgyi, Gábor Nagy, Tamás Papp

**Affiliations:** 1 MTA-SZTE Fungal Pathogenicity Mechanisms Research Group, Hungarian Academy of Sciences—University of Szeged, 6726 Szeged, Hungary; ibragimova_sandu@mail.ru (S.I.); szebecsilla@gmail.com (C.S.); csaba@bio.u-szeged.hu (C.V.); nagygab86@gmail.com (G.N.); 2 Department of Microbiology, Faculty of Science and Informatics, University of Szeged, 6726 Szeged, Hungary; homamoni@gmail.com; 3 Department of Genetics, Faculty of Science and Informatics, University of Szeged, 6726 Szeged, Hungary; rsinka@bio.u-szeged.hu (R.S.); alzyoudelham@gmail.com (E.I.A.)

**Keywords:** mucormycosis, gene disruption, uracil auxotrophy, OMP decarboxylase, non-homologous end joining, Mucorales

## Abstract

*Lichtheimia corymbifera* is considered as one of the most frequent agents of mucormycosis. The lack of efficient genetic manipulation tools hampers the characterization of the pathomechanisms and virulence factors of this opportunistic pathogenic fungus. Although such techniques have been described for certain species, the performance of targeted mutagenesis and the construction of stable transformants have remained a great challenge in Mucorales fungi. In the present study, a plasmid-free CRISPR-Cas9 system was applied to carry out a targeted gene disruption in *L. corymbifera*. The described method is based on the non-homologous end-joining repair of the double-strand break caused by the Cas9 enzyme. Using this method, short, one-to-five nucleotide long-targeted deletions could be induced in the orotidine 5′-phosphate decarboxylase gene (*pyrG*) and, as a result, uracil auxotrophic strains were constructed. These strains are applicable as recipient strains in future gene manipulation studies. As we know, this is the first genetic modification of this clinically relevant fungus.

## 1. Introduction

Mucormycosis is an invasive opportunistic fungal infection caused by certain members of the filamentous fungal order Mucorales [1]. This life-threatening infection can manifest as rhino-orbito-cerebral, pulmonary, cutaneous, gastrointestinal, or disseminated disease and is notorious for its rapid progression and unacceptably high mortality rates (i.e., around 50%, but 90% for disseminated cases) [2,3,4]. It most frequently occurs in patients having an underlying immunocompromised status due to immunosuppressive treatment or hematological malignancy but diabetes, especially if associated with ketoacidosis, elevated levels of free iron in the blood, critical care and trauma are also considered as possible risk factors [1,2,5,6]. The species most frequently identified as the etiological agents of mucormycosis belong to the genera *Rhizopus*, *Lichtheimia,* and *Mucor* [5,7,8]. After *Rhizopus delemar* (sometimes also referred to as *R. arrhizus* var. *delemar* and *R. oryzae*) [9], the *Lichtheimia* species, primarily *L. corymbifera*, are most often recovered from clinical samples, especially in European countries [2,4,8].

The frequency of systemic mucormycosis is increasing, mostly because of the broadening of the susceptible population [3,8,10]. Successful outcome highly depends on the timely diagnosis and the effective antifungal therapy, as well as the reversal of the predisposing factors [1] but, unfortunately, diagnosis and treatment of such infections still remain challenging [11]. Furthermore, Mucorales fungi display intrinsic resistance to the majority of the routinely used antifungal agents (e.g., candins and most azoles), which also limits the possible therapeutic options [1,3]. All these considerations urge the improvement of the molecular identification methods and the discovery of new antifungal targets and strategies. To achieve these goals, clarification of the pathomechanism of mucormycosis, understanding the interaction of these fungi with the hosts, and the identification of potential virulence factors and new biomarkers are essential. All these studies need the adaptation and routine application of molecular and genetic manipulation methods.

For *Rhizopus* and *Mucor* species, various classical genetic transformation-based molecular manipulation methods have been developed and applied [12,13]. Recently, the CRISPR-Cas9 method has also been adapted to generate single and multiple gene disruption in *Mucor circinelloides* [14,15] and point mutations in *Rhizopus delemar* [11]. However, until now, no effective genetic transformation methods have been available for *L. corymbifera*, which seriously hampers the efforts to characterize the gene functions and reveal the genetic and molecular background of the pathogenicity of this fungus. Therefore, a plasmid-free, in vitro CRISPR-Cas9 method has been adapted and tested to carry out targeted gene disruption in *L. corymbifera* in this study. Using this technique, the *pyrG* gene encoding the orotidine 5′-phosphate decarboxylase (OMP decarboxylase) has been disrupted and a uracil auxotrophic strain, which can be used as a recipient strain in further genetic manipulation studies, could be created.

## 2. Results

### 2.1. Disruption of the pyrG Gene in L. corymbifera

Two guide RNAs (gRNAs) targeting different positions in the *L. corymbifera pyrG* gene were designed and synthesized in vitro (Table 1). *L. corymbifera* protoplasts were co-transformed directly with one of these gRNAs and the Cas9 nuclease.

**Figure 1:**
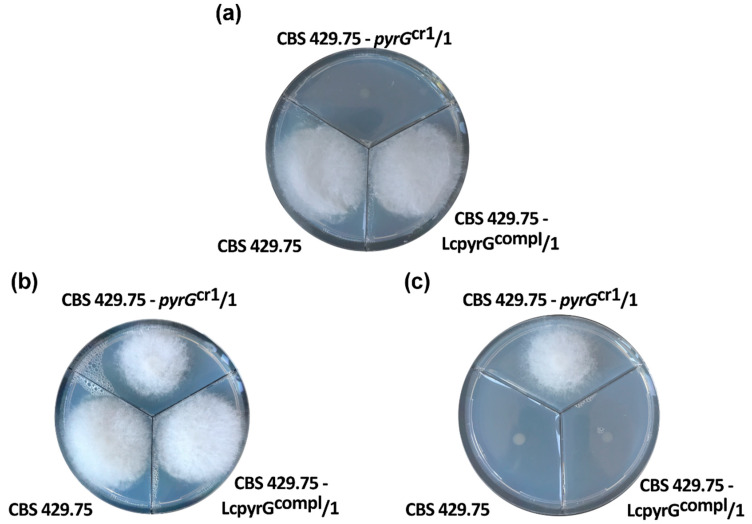
An example for the growth of the wild-type and the mutant *L. corymbifera* strains on yeast nitrogen base (YNB) medium (**a**), YNB medium supplemented with uracil (0.5 g/L) (**b**) and YNB medium supplemented with uracil (0.5 g/L) and 5-FOA (1 g/L) (**c**). Strains: CBS 429.75, the original wild-type strain; CBS 429.75–pyrG^Cr1^/1, one of the mutants, in which the *pyrG* was disrupted by the CRISPR-Cas9 method; CBS 429.75–*pyrG*^compl^/1, the strain, in which the CRISPR-Cas9-generated mutation was complemented. Growth was performed at 37 °C for two days.

Transformants, in which the targeted *pyrG* gene had been disrupted, were selected for their resistance to 5-fluoroorotic acid (5-FOA). Cells expressing OMP decarboxylase convert 5-FOA to a toxic compound, 5-fluorouridine monophosphate and cannot grow if 5-FOA is added to their culture medium [16,17]. Thus, colonies, which were unable to express the enzyme because of the mutation of the *pyrG* gene, display 5-FOA resistance and are auxotrophic to uracil (Figure 1). Using 10-10 µM gRNA and Cas9, the transformation efficiency was 8 colonies per 10^5^ protoplasts for both gRNAs.

### 2.2. Evidence for CRISPR-Cas9-Mediated Disruption of the pyrG Gene

**Figure 2:**
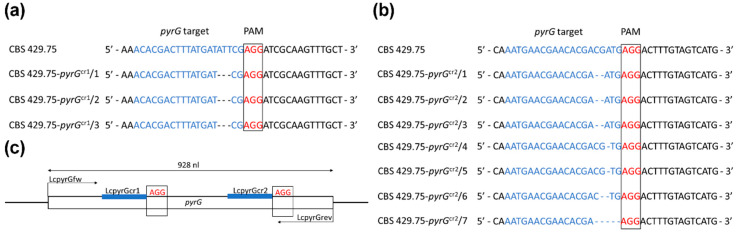
CRISPR-Cas9 induces deletions in the targeted sites of the *L. corymbifera pyrG* gene. (**a**) Sequence of the site in the *pyrG* gene targeted using the LcpyrGcr1 protospacer and location of the induced mutations in the three resulting strains. (**b**) Sequence of the site in the *pyrG* gene targeted using the LcpyrGcr2 protospacer and location of the induced mutations in the seven resulting strains. The protospacer and the protospacer adjacent motif (PAM) sequence are highlighted with blue and red letters, respectively. (**c**) Diagram representing the *pyrG* gene and showing the positions of the targeted sites and the primers used to analyze the mutants.

The *pyrG* gene was amplified from the 16 isolated 5-FOA resistant and uracil auxotrophic strains. Sequencing of the PCR products indicated one to five nucleotides long deletions at the targeted sites (Figure 2) in 10 transformants. These 10 isolates were named as CBS 429.75–*pyrG*^cr1^/1–3 and CBS 429.75–*pyrG*^cr2^/1–7, corresponding to the protospacers LcpyrGcr1 and LcpyrGcr2, respectively. No mutations were found in the sequences of the fragments amplified from the other six strains. This result indicates a genome editing efficiency of 37.5% and 87.5% for the protospacers LcpyrGcr1 and LcpyrGcr2, respectively.

To test their mitotic stability, each disruption mutant was passed several times on selective and non-selective media. All strains proved to be mitotically stable, retaining their phenotype (i.e., 5-FOA resistance and uracil auxotrophy) and the deletion in the *pyrG* gene after 5 cultivation cycles under non-selective conditions.

**Figure 3:**
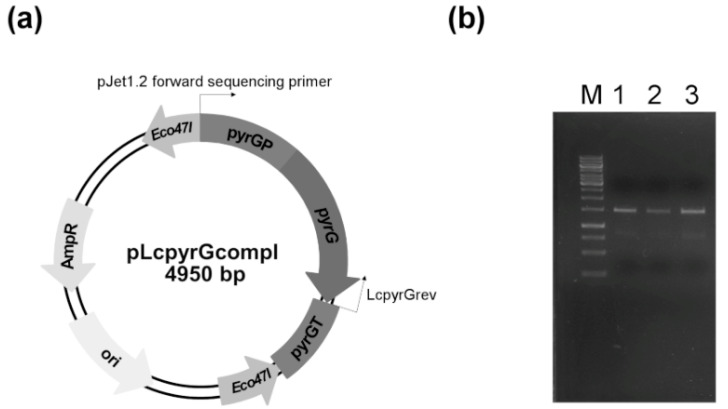
(**a**) Map of the plasmid used to complement the uracil auxotrophy of the CRISPR-Cas9-generated *L. corymbifera* mutants. Arrows indicate the recognition sites of the primers used to analyze the transformants. (**b**) An example of the PCR analysis of the complemented mutants. A fragment of the transferred plasmid was amplified from the *pyrG*-complemented CBS 429.75–pyrG^cr1^/1 strain using the primers pJet1.2 forward sequencing primer and LcpyrGrev. Lane M: GeneRuler 1 kb DNA Ladder (Thermo Scientific), Lane 1: CBS 429.75–*pyrG*^compl^/1, Lane 2: CBS 429.75–*pyrG*^compl^/2, Lane 3: CBS 429.75–*pyrG*^compl^/3.

Transformation of the mutants with the circular plasmid pLcpyrGcompl, which assures the expression of the *pyrG* gene, complemented the uracil auxotrophy and caused sensitivity to 5-FOA in the transformants (Figure 1). The plasmid and the PCR analysis of the complemented strains to prove the transformation are shown in Figure 3.

### 2.3. Characterization of the Disruption Mutants

**Figure 4:**
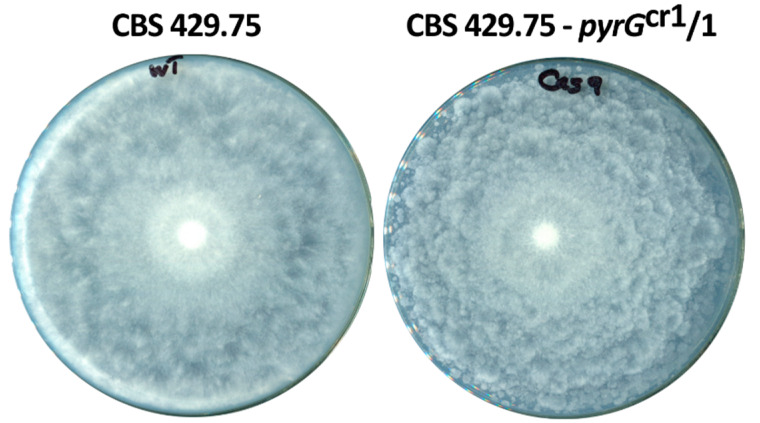
Colony morphology of the original CBS 429.75 and one of the mutants (CBS 429.75–*pyrG*^cr1^/1) growing on YNB plates at 37 °C.

Apart from their slightly weaker growth, the morphology of the mutants did not differ from that of the original strain (Figure 4).

**Figure 5:**
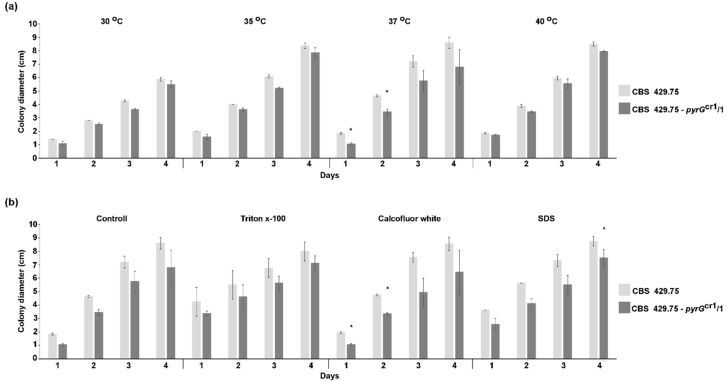
Growth of the original *L. corymbifera* strain (CBS 429.75) and one of the mutants (CBS 429.75–*pyrG*^cr1^/1) at different temperatures (**a**) and in the presence of different stressors (**b**). The presented values are averages; colony diameters were measured during three independent cultivation (error bars indicate standard deviation). Asterisk (*) indicates significant difference from the corresponding value of the CBS 429.75 strain according to the paired *t*-test (*p* < 0.05).

The growth ability of the mutants was tested, cultivating them on minimal medium supplemented with uracil at different temperatures for four days and measuring the colony diameters daily. Although the *pyrG* mutants displayed a tendency to grow slightly weaker than the parental strain, this difference in their growth did not prove to be significant, except at 37 °C after one day of cultivation (Figure 5a).

Effect of calcofluor white (CFW), sodium dodecyl sulfate (SDS), and Triton X-100 as cell-wall and membrane-perturbing compounds on the fungal growth was tested. In general, *pyrG* disruption mutants displayed somewhat weaker growth than the original strain in the presence of these compounds (Figure 5b).

**Figure 6:**
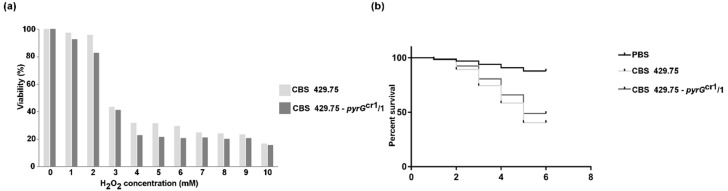
(**a**) Susceptibility of the original *L. corymbifera* strain (CBS 429.75) and one of the mutants (CBS 429.75–*pyrG*^cr1^/1) to H_2_O_2_. (**b**) Virulence of the strains CBS 429.75 and CBS 429.75–*pyrG*^cr1^/1 in *Drosophila* survival experiments.

Susceptibility of the *pyrG* disruption mutants and the original strain to oxidative stress was also examined. Strains, in which the *pyrG* gene was disrupted, showed a tendency to slightly higher sensitivity to hydrogen peroxide (H_2_O_2_) than the parental strain, but this difference was not found to be significant (Figure 6a).

Pathogenicity of the mutants and the original strain did not differ significantly in survival experiments using the alternative *Drosophila melanogaster* model (Figure 6b).

## 3. Discussion

*L. corymbifera* is considered as the second most frequent agent of mucormycosis after *R. delemar*, especially in Europe [2,4,8]. However, efficient genetic transformation or genome edition methods have not been available for this fungus, which highly narrows the spectrum of tools applicable in genetic and molecular studies of the pathogenicity. At the same time, it is also known that targeted gene manipulation is a great challenge in the Mucorales group, and such methods have been developed only for certain species, such as *M. circinelloides* [11,12,13,14].

Application of the CRISPR-Cas9 system provides a versatile genome editing technique, which has already been used to target and disrupt genes in some pathogenic fungi, such as in *Aspergillus fumigatus* [18], *Candida albicans* [19], *C. glabrata* [20] or *Cryptococcus neoformans* [21] Recently, the method has also successfully been adapted to two members of Mucorales, *M. circinelloides* [14,15] and *R. delemar* [11].

Various methods have been developed to carry out the CRISPR-Cas9-based genome editing in fungi [22]. In vivo expression systems involve the application of plasmids, which assure the expression of the Cas9 enzyme and the elements of the gRNA, i.e., the CRISPR-RNA (crRNA) containing the protospacer sequence and the trans-activating crRNA (tracrRNA). This strategy was used to induce targeted point mutations in the genome of *R. delemar* and construct a uracil auxotrophic strain [11]. It should be mentioned that in vivo systems may have the drawbacks of time-consuming plasmid construction, the possible presence of heterologous sequences in the genome-edited strains (such as bacterial antibiotic resistance genes) and maintenance of the plasmid and expression of the system after the genome edition event, which may cause off-target effect or other interference between the transferred DNA and the host genome [14].

In vitro CRISPR-Cas9 systems use a plasmid-free strategy where the purified Cas9 enzyme and the in vitro transcribed gRNA complex is transferred together into the recipient cells. Application of such in vitro systems can minimalize the chance for the emergence of the off-target effect and assure the rapid degradation of the CRISPR-Cas9 ribonucleoprotein complex after the genetic modification [23]. This approach was successfully applied to construct single and multiple gene disruption mutants in *P. chrysogenum* [24] and *M. circinelloides* [14,15]. This in vitro, plasmid-free strategy has been adapted to achieve stable, targeted gene disruption in *L. corymbifera* in the present study.

Cas9 causes double-strand breaks at the targeted site, which is repaired by either the non-homologous end joining (NHEJ) or the homology-directed repair (HDR) mechanisms of the cell. The latter requires the presence of a template DNA containing sequences homologous with the targeted site to drive the homologous recombination. NHEJ generally causes indel (insertion or deletion) mutations and can be used for targeted gene disruption. These indel mutations are generally short with lengths ranging from one to a few tens of nucleotides in various fungi [11,18,22,25]. In our study, NHEJ repair induced various 1–5 nucleotides long deletions in the *pyrG* gene of *L. corymebifera*. Previously, the same mechanism was used to induce a 1-nucleotide deletion in the *pyrF* gene of *R. delemar* [11]. Interestingly, NHEJ caused extensive, more than 2 kbp long, deletions in the targeted *carB* and the adjacent genes of *M. circinelloides*, raising that HDR can be the preferred approach for the CRISPR-Cas9 genome editing of that fungus [14].

Although 16 5-FOA resistant *L. corymbifera* strains were isolated, 10 of them harbored deletions at the targeted site as a consequence of the CRISPR-Cas9 mutagenesis. 5-FOA resistance can be used to select for mutants in the genes *pyrF* encoding orotate phosphoribosyl transferase and *pyrG* encoding OMP decarboxylase [12,16]. Both genes participate in the uridine biosynthesis pathway and their deletion causes uracil auxotrophy. For the two genes, uracil auxotrophic and 5-FOA resistant mutants can emerge spontaneously [11,16]. Such spontaneous mutations, possibly in the *pyrF* gene, may have caused the six false-positive colonies.

Besides demonstrating the applicability of the CRISPR-Cas9 methods for the genetic modification of *L. corymbifera*, another aim of the study is to construct a stable uracil auxotrophic mutant, which can be applied in further gene manipulation and genetic transformation studies as a recipient strain. Using this strain, the *pyrG* gene of *L. corymbifera* can be applied as a selectable marker. Uracil auxotrophy may cause decreased growth and pathogenicity, and several studies have demonstrated that the pyrimidine biosynthetic pathway can affect these processes [26,27,28,29]. Recently, Binder et al. [30] reported that the uracil auxotrophy of *M. circinelloides* caused decreased virulence in the alternative *Galleria* model. However, uracil or uridine availability and activity of the uptake mechanisms can modify these effects in the different species and strains [31]. Therefore, growth ability, stress tolerance, and virulence were tested in the mutants. In general, disruption of the *pyrG* gene had a moderate effect on the viability of the mutants. It did not affect the morphology and the pathogenicity of the fungus and slightly decreased its growth at certain temperatures and under cell wall stress. H_2_O_2_ may induce biophysical and permeability changes in the cell membrane [32], potentially affecting the uracil uptake in the auxotrophic strains. However, the H_2_O_2_ sensitivity of the mutants only slightly differed from those of the parental strain. At the same time, complementation of the disrupted *pyrG* gene restored the original phenotype. These results indicate that the *pyrG* mutants can be used as recipient strains in gene manipulation and other functional genetic experiments.

## 4. Materials and Methods

### 4.1. Strains, Media and Growth Conditions

The *L. corymbifera* type-strain (CBS 429.75) was used in this study. For nucleic acid extractions, 10^6^ sporangiospores were plated onto a solid minimal medium (YNB; 10 g glucose, 0.5 g yeast nitrogen base without amino acids (BD Difco, Becton Dickinson, Franklin Lakes, NJ, USA), 1.5 g (NH_4_)_2_SO_4_, 1.5 g sodium glutamate, and 20 g agar per liter) supplemented with uracil (0.5 mg/mL), if required. Fungal cultures were grown for 4 days at 37 °C. To examine the effect of the temperature on fungal growth, strains were cultivated on solid YNB supplemented with uracil at 30, 35, 37, and 40 °C, plating 10^4^ spores onto the medium. To test the mitotic stability of the transformants, malt extract agar (MEA; 10 g glucose, 5 g yeast extract, 10 g malt extract, and 20 g agar per liter) was used as the complete, non-selective medium.

### 4.2. Molecular Techniques and Design of the gRNA

Genomic DNA was isolated using the ZR Fungal/Bacterial DNA MiniPrep (Zymo Research, Irvine, CA, USA) according to the manufacturer’s instructions. PCR products were isolated and concentrated using the Zymoclean Large Fragment DNS Recovery Kit (Zymo Research, Irvine, CA, USA) and DNA Clean & Concentrator-5 (Zymo Research, Irvine, CA, USA). To design the oligonucleotide sequences, sequence data available in the *Lichtheimia corymbifera* JMRC:FSU:9682 genome [33] database at the JGI MycoCosm portal (https://mycocosm.jgi.doe.gov/Liccor1/Liccor1.home.html) were used. Two different protospacer sequences were designed to target the DNA cleavage in the *pyrG* gene (*Lichtheimia corymbifera* JMRC:FSU:9682 genome database ID: LCOR_02455.1) and named as LcpyrGcr1 and LcpyrGcr2. The sequences and positions of the corresponding regions in the genome are presented in Table 1. Using these sequences, the crRNA and the tracrRNA molecules were purchased from IDT as Alt-R CRISPR crRNA and Alt-R CRISPR-Cas9 tracrRNA, respectively. To form the crRNA:tracrRNA duplex (i.e., the gRNA), the Nuclease-Free Duplex Buffer (IDT, Coralville, IA, USA) was used according to the instructions of the manufacturer. To prove the mutations generated in the transformants, the *pyrG* gene was amplified by PCR using the Phusion Flash High-Fidelity PCR Master Mix kit (Thermo Scientific, Waltham, MA, USA) and the primer pair LcpyrGfw (5′-atgaacaccttcaagacatacag-3′) and LcpyrGrev (5′-ctactgcttttgcacacgttc-3′). The amplified fragment was then sequenced commercially by LGC Genomics (Berlin, Germany).

### 4.3. Transformation

The polyethylene glycol (PEG)-mediated protoplast transformation was used to introduce one of the gRNAs and the Cas9 enzyme (Alt-R S.p. Cas9 Nuclease; IDT, Coralville, IA, USA) simultaneously into the *L. corymbifera* cells as described earlier for *M. circinelloides* [14]. Ten μM gRNA and 10 μM Cas9 nuclease were added to the protoplasts in one transformation reaction. Transformants were selected on solid YNB medium supplemented with 0.8 M sorbitol as an osmotic stabilizer, 0.5 g/L uracil, and 1 g/L 5-FOA (Thermo Scientific, Waltham, MA, USA).

### 4.4. H_2_O_2_ Susceptibility Tests

The sensitivity of the fungal strains to H_2_O_2_ was examined in a 96-well microtiter plate assay. H_2_O_2_ solution (Sigma Aldrich, St. Louis, MO, USA) was dissolved in liquid YNB medium to prepare a 100-mM stock solution. Final concentrations of H_2_O_2_ in the wells ranged from 0 to 10 mM; chemicals were diluted with liquid YNB medium. Spore suspensions were also prepared in liquid YNB (supplemented with 0.5 g/L uracil, if required), and the final amount of the spores in the wells was set to 10^5^. Plates were incubated for 48 h at 37 °C and the optical density of the fungal cultures was measured at 620 nm using a SPECTROstar Nano (BMG Labtech, Ortenberg, Germany) microplate reader. The uninoculated medium was used as the background for the calibration, and fungal growth in the H_2_O_2_-free medium was considered as 100%; all experiments were performed in triplicates.

### 4.5. Effect of Cell Wall Stressors

Fungal spores (10^4^/mL) were point-inoculated at the center of solid YNB media (supplemented with 0.5 g/L uracil, if required) containing 1 µL/mL Triton-X 100 (Sigma Aldrich, St. Louis, MO, USA), 0.04 mg/mL SDS (Sigma Aldrich, St. Louis, MO, USA) or 0.1 mg/mL CFW (Sigma Aldrich, St. Louis, MO, USA) as the tested stressors. The diameter of the colonies was measured daily after incubating the plates at 37 °C.

### 4.6. Survival Experiments in Drosophila melanogaster

Pathogenicity of the original CBS 429.75 *L. corymbifera* strain and one of the constructed mutants (CBS 429.75-*pyrG*^cr1^/1) was tested in *D. melanogaster*. Spore suspensions were prepared with sterile phosphate buffer saline (PBS; 137 mM NaCl, 2.7 mM KCl, 10 mM Na_2_HPO_4_, 2 mM KH_2_PO_4_, pH 7.4) from 4-day-old cultures grown on YNB plates (supplemented with 0.5 g/L uracil, if required) at 37 °C. The final inoculum concentrations were adjusted to 1 × 10^7^ spores/mL with PBS. *Drosophila* stocks were raised and kept following the infection on standard cornmeal agar medium at 25 °C. The Oregon R strain, originally obtained from the Bloomington stock center, was used as the wild-type throughout the experiments. Infection was performed by dipping a thin needle in a suspension of fungal conidia (10^7^ conidia/mL) or PBS for the uninfected control, and, subsequently, the thorax of the anesthetized fly was pricked. Flies were counted at different points of time to monitor survival. Flies were moved into fresh vials every other day. Each experiment was performed with approximately 60 flies for each genotype. The results shown are representative of at least three independent experiments.

### 4.7. Construction of Plasmids for the Complementation of Uracil Auxotrophy

The *pyrG* gene with its own promoter and terminator sequences was amplified by PCR using the Phusion Flash High-Fidelity PCR Master Mix (Thermo Scientific, Waltham, MA, USA) and the primer pair LcpyrG1 (5′-gtaatagcaaggaccaccgagtga-3′) and LcpyrG2 (5′-gaacaattaagagccgttgaatcc-3′). The amplified fragment was ligated into the pJet1.2 cloning vector (Thermo Scientific, Waltham, MA, USA), arising the plasmid pLcpyrGcompl (Figure 3a), which was used to complement the uracil auxotrophy of the *pyrG* disrupted mutants. The plasmid was transformed into the mutants by PEG-mediated protoplast transformation. Transformants were selected on YNB plates without the addition of uracil. One transformant colony was isolated and analyzed for each mutant. The presence of the plasmids and the *pyrG* gene in the transformants were proven by PCR using the pJet1.2 forward sequencing primer (5′-cgactcactatagggagagcggc-3′), which has the binding site on the plasmid, and the LcpyrGrev primer (Figure 3b).

### 4.8. Statistical Analyses

All measurements were performed in at least two technical and three biological replicates. Significance was calculated with paired *t*-test using Microsoft Excel of the Microsoft Office package (Microsoft, Redmond, WA, USA). P-values less than 0.05 were considered statistically significant.

## 5. Conclusions

A plasmid-free, in vitro CRISPR-Cas9 system was adopted to carry out targeted mutagenesis in *L. corymbifera*. Our results demonstrate that the CRISPR-Cas9 method based on NHEJ is applicable to induce short, targeted deletions in the genes of this fungus. Using the described technique, the *pyrG* gene encoding the OMP decarboxylase has been disrupted. The resulting uracil auxotrophic *L. corymbifera* strains can be applied as recipient strains in further genetic manipulation studies. As we know, this is the first targeted gene manipulation of this mucormycosis-causing fungus.

## Figures and Tables

**Table 1:** Sequences of the protospacers designed in the study and positions of the corresponding regions in the *Lichtheimia corymbifera pyrG* gene.

Designation	Sequence (5′–3′)	Position in the *pyrG* Gene
LcpyrGcr1	acacgactttatgatattcg	315–334 **^1^**
LcpyrGcr2	aatgaacgaacacgacgatg	687–707

^1^ Numbers present nucleotide positions downstream from the start codon of the *L. corymbifera pyrG* gene (gene ID: ID: LCOR_02455.1).

## Abbreviations

5-FOA	5-fluoroorotic acid
Cas9	CRISPR-associated protein 9
CFW	calcofluor white
CRISPR	clustered regularly interspaced short palindromic repeats
crRNA	CRISPR-RNA
gRNA	guide RNA
H_2_O_2_	hydrogen peroxide
HDR	Homology-directed repair
MEA	malt extract agar
NHEJ	non-homologous end-joining
OMP	orotidine 5′-phosphate
PAM	protospacer adjacent motif
PBS	phosphate buffer saline
PEG	polyethylene glycol
*pyrF*	orotate phosphoribosyl transferase gene
*pyrG*	orotidine 5′-phosphate decarboxylase gene
SDS	sodium dodecyl sulfate
tracrRNA	trans-activating crRNA
YNB	yeast nitrogen base
